# Heritable Gut Microbiome Associated with *Salmonella enterica* Serovar Pullorum Infection in Chickens

**DOI:** 10.1128/mSystems.01192-20

**Published:** 2021-01-05

**Authors:** Jinmei Ding, Hao Zhou, Lingxiao Luo, Lu Xiao, Kaixuan Yang, Lingyu Yang, Yuming Zheng, Ke Xu, Chuan He, Chengxiao Han, Huaixi Luo, Chao Qin, Fisayo T. Akinyemi, Caiju Gu, Zhenxiang Zhou, Qizhong Huang, He Meng

**Affiliations:** a Shanghai Key Laboratory of Veterinary Biotechnology, Department of Animal Science, School of Agriculture and Biology, Shanghai Jiao Tong University, Shanghai, People’s Republic of China; b Animal Husbandry and Veterinary Research Institute, Shanghai Academy of Agricultural Science, Shanghai, People’s Republic of China; University of Georgia

**Keywords:** heritable, host genetic variants, gut microbiota, mGWAS, *Salmonella* Pullorum, chicken

## Abstract

Pullorum disease is one of the most common diarrhea-related diseases caused by Salmonella enterica subspecies *enterica* serovar Gallinarum biovar Pullorum (*S*. Pullorum); it negatively affects the poultry industry. However, limited studies have explored the association between the gut microbiota and *S*. Pullorum infection in chickens. In the present study, we performed a microbiome comparison and a microbiome genome-wide association study (mGWAS) to investigate the association among the host genetics, the gut microbiota, and pullorum disease in chickens. We found that *S*. Pullorum infection in chickens could alter the abundance of 39 bacterial genera (*P < *0.05). The altered structure and composition of the gut microbiota were also detected in the offspring. mGWAS results revealed host genetic variants to be prominently associated with gut microbial diversity and individual microbes. The pathogens *Pelomonas* and *Brevundimonas*, which had a high abundance in positive parent chickens and their offspring, were significantly associated with several genetic mutations in immunity-related genes, such as *TGIF1*, *TTLL12*, and *CCR7*. This finding explained why *Pelomonas* and *Brevundimonas* were heritable in *S*. Pullorum-infected chickens. The heritable gut microbes and identified genetic variants could provide references for the selection of resistant chickens and the elimination of pullorum disease.

**IMPORTANCE** The present study investigated the association among the host genome, the gut microbiome, and *S*. Pullorum infection in chickens. The results suggested that the gut microbial structure is altered in *S*. Pullorum-infected chickens. The diversity and abundance of the gut microbiota remarkably differed between the offspring coming from *S*. Pullorum-positive and *S*. Pullorum-negative chickens. Heritable gut microbiota were detected in the offspring. Moreover, host genetic variants were associated with microbial diversity and individual gut microbes. The pathogens *Pelomonas* and *Brevundimonas*, which exhibited a high heritability in *S*. Pullorum-positive parents and their offspring, were associated with several genetic mutations in immunity-related genes.

## Abstract (precis)

The present study investigated the association among the host genome, the gut microbiome, and *S*. Pullorum infection in chickens. The results suggested that the gut microbial structure is altered in *S*. Pullorum-infected chickens.

## INTRODUCTION

Pullorum disease is an acute systemic disease specific to poultry. The disease mainly occurs in young chicks, causing white diarrhea with high mortality. It is caused by Salmonella enterica subspecies *enterica* serovar Gallinarum biovar Pullorum (*S*. Pullorum) (1, 2). Since the early 20th century, pullorum disease has caused substantial economic losses in the poultry industry (3, 4). This disease has a higher mortality rate in 2- to 3-week-old chicks. It rarely occurs in adult birds, and only some infected adult birds show the symptoms of weight loss, diarrhea, inappetence, lesions, and reproductive tract abnormalities (5). Pullorum disease is widely spread and is difficult to cure because of the vertical and horizontal transmission of *S*. Pullorum (6). Some infected chickens are asymptomatic carriers and can transmit the bacteria to their offspring and other chickens in the flock (7). In 1927, Runnells et al. developed a rapid slide agglutination test based on the *S*. Pullorum antigen antibody reaction to eliminate infected individuals (8). Although a strict eradication program has been implemented for the *S*. Pullorum-infected chicken population and some success has been achieved (9, 10), the outbreaks of pullorum disease in chickens indicate that *S*. Pullorum infection is still frequent and results in considerable economic losses in the poultry industry (11–14). This is mainly because the eradication program results are erratic, including false-negative reactions and a lack of sensitivity (15). Therefore, a new insight is needed to prevent *S*. Pullorum infection in chickens.

The variation of gut microbial composition and function could be linked to various diseases in mammals and birds, including obesity (16, 17), diarrhea (18, 19), cancer (20), and inflammatory bowel disease (21). In the case of acute inflammation triggered by enteric pathogens, such as *Salmonella*, the pathogens compete with the gut microbiota and overcome the host immune defenses (22). For example, *S*. Typhimurium can overcome colonization resistance by abusing the host’s inflammatory immune response to gain an edge over the normal gut microbial community (23, 24). Moreover, *S*. Pullorum challenge has been found to induce ileal inflammation mediated by proinflammatory cytokines and influence the abundance and diversity of ileal microbes in laying hens (25). Chickens with various genetic backgrounds exhibit various levels of resistance to *Salmonella* (26). The susceptibility or resistance to *Salmonella* is related to the host genetics (27, 28). Several studies have discovered candidate genes associated with the death and carrier state of chickens after *Salmonella* infection (29, 30). Our previous study revealed 43 host genetic markers associated with *S*. Pullorum infection in chickens (31). These findings indicate that the resistance to *Salmonella* is closely associated with the gut microbiota and host genetic variants. The gut microbiome can be treated as phenotypes in microbiome genome-wide association studies (mGWAS) to explore the interaction between the microbiota and host genetic variants (32–34). However, few studies have examined chicken pullorum disease, a complex and vertically transmitted bacterial disease, from the perspective of host genetic variants and the gut microbiota.

**FIG 1:**
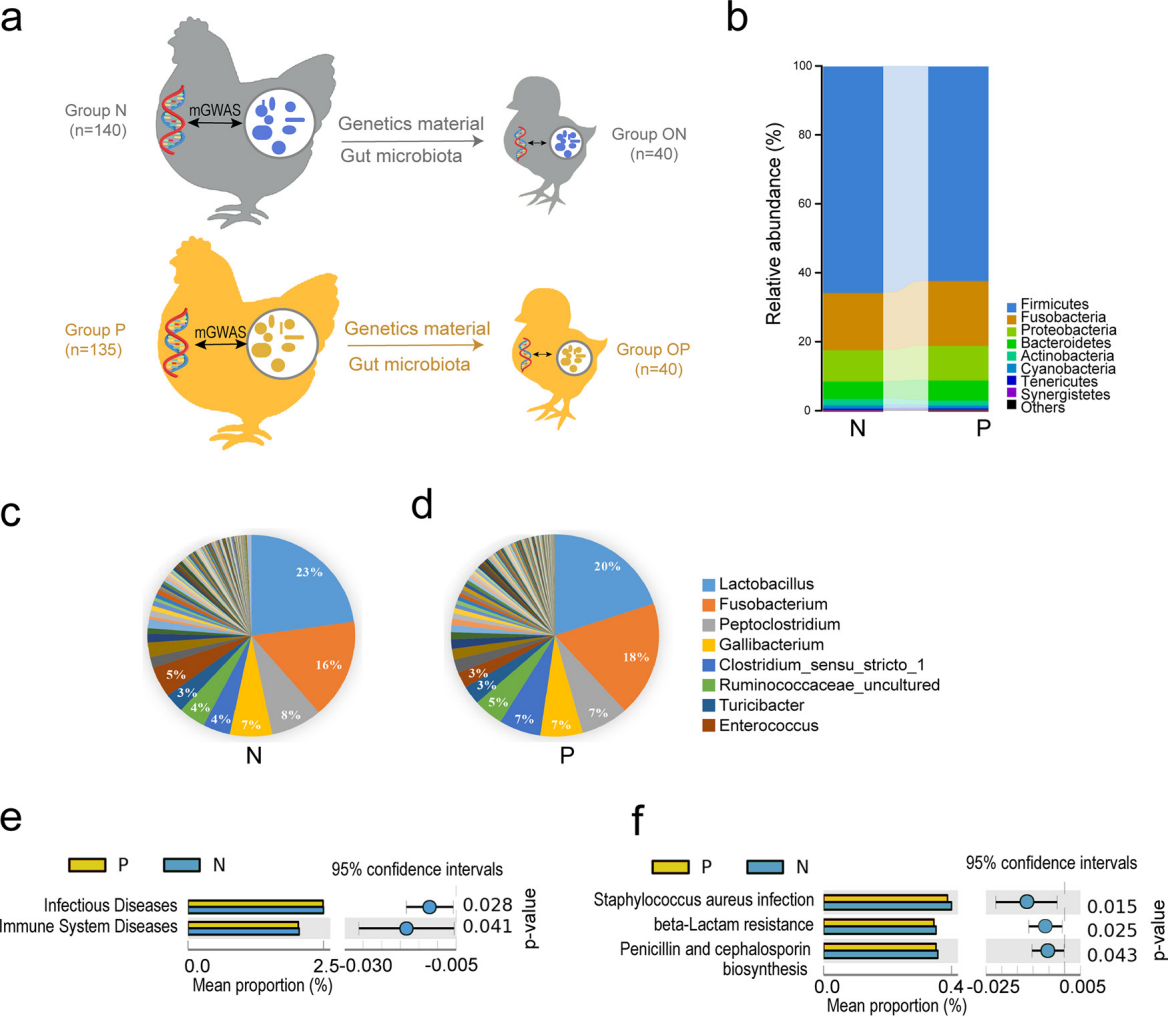
Gut microbial characteristics of *S*. Pullorum-negative (group N) and *S*. Pullorum-positive (group P) chickens. (a) Diagram of genetic material and gut microbial transmission from parents to offspring. (b) Comparison of the gut microbiota between groups P and N at the phylum level. (c) Gut microbial composition of group N at the genus level. (d) Gut microbial composition of group P at the genus level. Only the major taxonomic groups are shown. (e) Comparison of the microbial functional pathways between groups N and P at KEGG level two. (f) Comparison of the microbial functional pathways between groups N and P at KEGG level three.

In the present study, we performed a microbiome comparison and mGWAS to investigate the association among the host genetics, the gut microbiota, and pullorum disease in chickens (Fig. 1a). Microbiome comparison between *S*. Pullorum-negative and *S*. Pullorum-positive chickens (groups N and P, respectively) and their respective offspring (groups ON and OP, respectively) was carried out to assess the association between the gut microbial composition and *S*. Pullorum infection. mGWAS was used to evaluate the contribution of host genomic loci to microbial beta diversity and the abundance of individual microbes. Our discovery provides more information to identify heritable gut microbiota and potential genetic loci associated with *S*. Pullorum infection and could help in the elimination of infected chickens and the selection of resistant chickens.

## RESULTS

### *S*. Pullorum infection altered the gut microbial characteristics of chickens.

To examine the effect of *S*. Pullorum infection on the gut microbiome of chickens, the microbial composition in groups N and P was compared. Nineteen phyla were detected in the two groups. The dominant phyla were *Firmicutes* (65.5% in group N and 62.1% in group P), *Fusobacteria* (16.3% in group N and 18.7% in group P), and *Proteobacteria* (9.37% in group N and 9.95% in group P) (Fig. 1b). The preponderant genera were *Lactobacillus* (*Firmicutes*), *Fusobacterium* (*Fusobacteria*), *Peptoclostridium* (*Firmicutes*), and *Gallibacterium* (*Proteobacteria*) (Fig. 1c and d). Microbiota comparisons at the genus level revealed that the abundance of 39 genera differed between the two groups (*P < *0.05), with the difference being significant in the case of 33 out of 39 genera (*P < *0.01) (Table 1). *Klebsiella* (*Proteobacteria*), *Neisseria* (*Proteobacteria*), *Enhydrobacter* (*Proteobacteria*), *Leuconostoc* (*Firmicutes*), *Faecalibaculum* (*Firmicutes*), *Enterococcus* (*Firmicutes*), and *Mobilitalea* (*Firmicutes*) were enriched in group N, while *Anaerobiospirillum* (*Proteobacteria*), *Deinococcus* (*Deinococcus-Thermus*), *Phascolarctobacterium* (*Firmicutes*), *Brevundimonas* (*Proteobacteria*), *Pelomonas* (*Proteobacteria*), *Oscillibacter* (*Firmicutes*), and *Serratia* (*Proteobacteria*) were more abundant in group P than in group N. The metabolic pathways of Staphylococcus aureus infection, beta-lactam resistance, and penicillin and cephalosporin biosynthesis (related to infectious diseases and the biosynthesis of other secondary metabolites) were more enriched in group N than in group P (*P < *0.05) (Fig. 1e and f).

**TABLE 1:** Gut microbiota with significant differences between groups P and N at the genus level

Genus (phylum)	Mean difference		*P* value^*a*^	q value
	Group P	Group N		
*Aerococcus* (*Firmicutes*)	0.000429	0.000039	0**	0
*Alkalibacterium* (*Firmicutes*)	0.000003	0.000063	0**	0
*Anaerobiospirillum* (*Proteobacteria*)	0.000171	0.000076	0**	0
*Deinococcus* (*Deinococcus-Thermus*)	0.000074	0.000028	0**	0
*Dietzia* (*Actinobacteria*)	0.000001	0.000035	0**	0
*Faecalibaculum* (*Firmicutes*)	0.000038	0.000092	0**	0.000001
*Flaviflexus* (*Actinobacteria*)	0.000006	0.000038	0**	0.000001
*Fraxinus_excelsior *(*European_ash*) (*Cyanobacteria*)	0.000253	0.000048	0**	0
*Leuconostoc* (*Firmicutes*)	0.000002	0.000042	0**	0
*Neisseria* (*Proteobacteria*)	0.000002	0.000067	0**	0
*Oceanimonas* (*Proteobacteria*)	0	0.000079	0**	0
*Oceanisphaera* (*Proteobacteria*)	0.000008	0.000038	0**	0
*Oscillibacter* (*Firmicutes*)	0.000094	0.000036	0**	0
*Pelomonas* (*Proteobacteria*)	0.000161	0.000085	0**	0.000001
*Phascolarctobacterium* (*Firmicutes*)	0.000103	0.000044	0**	0
*Pisciglobus* (*Firmicutes*)	0.000598	0.000028	0**	0
*Serratia* (*Proteobacteria*)	0.000069	0.000008	0**	0
*Tissierella* (*Firmicutes*)	0.000002	0.000026	0**	0
*Victivallis* (*Lentisphaerae*)	0.000115	0.000071	0.00003**	0.000305
*Enhydrobacter* (*Proteobacteria*)	0.000003	0.00002	0.000034**	0.000325
*Mycoplasma* (*Tenericutes*)	0.000011	0.000029	0.000043**	0.000388
*Holdemania* (*Firmicutes*)	0.000064	0.000032	0.000052**	0.000451
*Candidatus_Sonnebornia_yantaiensis* (*Parcubacteria*)	0.000029	0.000063	0.000078**	0.000649
*Anaerococcus* (*Firmicutes*)	0.000014	0.000036	0.00013**	0.00104
*Lentibacillus* (*Firmicutes*)	0.000026	0.00005	0.000693**	0.0053
*Bosea* (*Proteobacteria*)	0.00004	0.000021	0.00149**	0.011
*Massilia* (*Proteobacteria*)	0.000093	0.000067	0.00165**	0.0117
*Rubrobacter* (*Actinobacteria*)	0.000033	0.000015	0.00172**	0.0118
*Ralstonia* (*Proteobacteria*)	0.000023	0.000009	0.00255**	0.0168
*Psychrobacter* (*Proteobacteria*)	0.000011	0.000028	0.0032**	0.0204
*Gelria* (*Firmicutes*)	0.000039	0.00002	0.0035**	0.0216
*Mobilitalea* (*Firmicutes*)	0.00005	0.000073	0.00383**	0.0229
*Bilophila* (*Proteobacteria*)	0.000031	0.000015	0.00711**	0.0412
*Micrococcus* (*Actinobacteria*)	0.000047	0.000026	0.0141*	0.0796
*Brevundimonas* (*Proteobacteria*)	0.000054	0.000028	0.017*	0.0929
*Enterorhabdus* (*Actinobacteria*)	0.000039	0.000027	0.0262*	0.138
*Enterococcus* (*Firmicutes*)	0.0307	0.0549	0.027*	0.138
*Klebsiella* (*Proteobacteria*)	0.00003	0.000045	0.0274*	0.138
*Lachnospira* (*Firmicutes*)	0.000011	0.000024	0.0486*	0.239

a *, *P *< 0.05; **, *P *< 0.01.

### *S*. Pullorum infection in chickens altered the offspring’s gut microbial composition.

**FIG 2:**
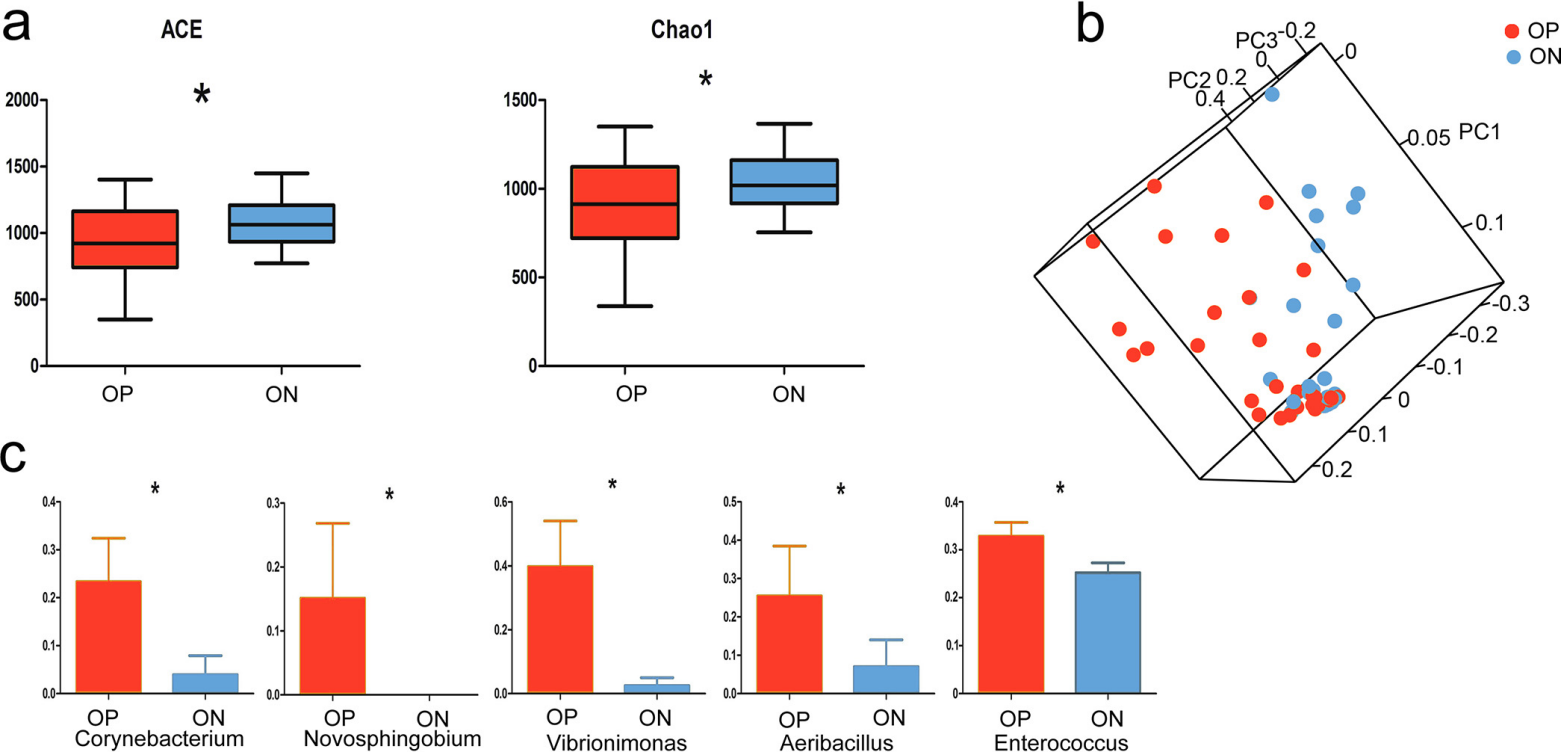
Gut microbial composition in offspring from *S*. Pullorum-positive parents and *S*. Pullorum-negative parents (groups OP and ON, respectively). (a) Microbial alpha diversity indices ACE and Chao1 between groups OP and ON (*, *P *< 0.05). (b) Microbial beta diversity in groups OP and ON with a principal component analysis (PCA) plot. (c) Comparison of the abundance of potentially harmful bacteria between groups OP and ON (*, *P* < 0.05).

To investigate the influence of the host genetics and *S*. Pullorum infection on the gut microbiota of the offspring, we compared the gut microbial composition between the offspring in groups OP and ON. The alpha diversity indices abundance-based coverage estimator (ACE) and Chao1 suggested that the community richness of group OP was remarkably lower than that of group ON (*P < *0.05) (Fig. 2a). By applying principal component analysis (PCA) to microbial beta diversity, the offspring could be classified into their respective groups (Fig. 2b). Of the 13 phyla detected, *Firmicutes*, *Proteobacteria*, and *Bacteroidetes* were the dominant phyla (see Fig. S1a in the supplemental material). The abundance of *Parcubacteria* was significantly enriched in group OP (*P < *0.01) (Table S1). At the genus level, *Lactobacillus* was more abundant in group ON (62%) than in group OP (53%), while the percentage of *Enterococcus*, *Fusobacterium*, and *Helicobacter* (*Proteobacteria*) was greater in group OP than in group ON (Fig. S1b). Forty-one genera were prominently different between the two groups (*P < *0.05) (Table S2). The abundance of potentially harmful bacteria, namely, *Corynebacterium* (*Actinobacteria*), *Novosphingobium* (*Proteobacteria*), *Vibrionimonas* (*Bacteroidetes*), *Aeribacillus* (*Firmicutes*), and *Enterococcus*, was higher in group OP than in group ON (*P < *0.05) (Fig. 2c). On the other hand, beneficial bacteria, such as *Kurthia* (*Firmicutes*), *Acidovorax* (*Proteobacteria*), and *Comamonas* (*Proteobacteria*), were abundant in group ON (Table S2). *Pelomonas* and *Brevundimonas*, which were enriched in group P, were more abundant in group OP than in group ON (Table S2).

10.1128/mSystems.01192-20.1 FIG S1 Gut microbial characteristics of groups OP and ON. Download FIG S1, TIF file, 0.9 MB. Copyright © 2021 Ding et al. 2021 Ding et al. This content is distributed under the terms of the Creative Commons Attribution 4.0 International license.

10.1128/mSystems.01192-20.2 TABLE S1 Gut microbiota with significant difference between groups OP and ON at the phylum level. Download Table S1, DOCX file, 0.01 MB. Copyright © 2021 Ding et al. 2021 Ding et al. This content is distributed under the terms of the Creative Commons Attribution 4.0 International license.

10.1128/mSystems.01192-20.3 TABLE S2 Gut microbiota with significant difference between groups OP and ON at the genus level. Download Table S2, DOCX file, 0.02 MB. Copyright © 2021 Ding et al. 2021 Ding et al. This content is distributed under the terms of the Creative Commons Attribution 4.0 International license.

### Host genetic loci associated with gut microbial beta diversity.

**FIG 3:**
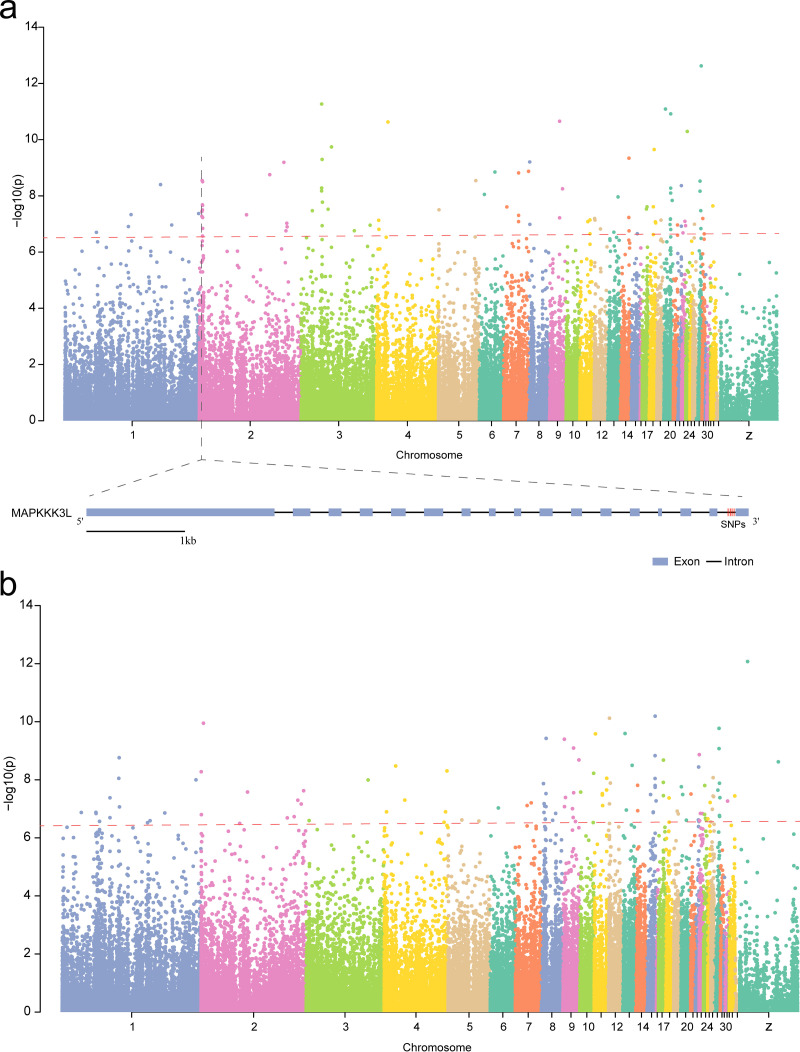
Host genetic variants associated with gut microbiota. A Manhattan plot of genome-wide associations between host genetic variants and gut microbial beta diversity in group P (a) and group N (b). SNPs above the red line were considered significant SNPs (*P < *3.1 × 10^−7^).

mGWAS was used to study the association between host genetics and gut microbial diversity. In total, 109 significant single-nucleotide polymorphisms (SNPs) (*P < *3.1 × 10^−7^) were identified in group P (Fig. 3a and Table S3). The most significant SNP is at 7,092,243 bp on Gallus gallus chromosome (GGA) 27, which is located at the intergenic region of *IKZF3* and *ZPBP2* (Table S3). Seventeen SNPs were clustered on GGA2 in group P (Fig. 3a). Of these, 4 SNPs were located at the intronic region of *MAPKKK3L* (Fig. 3a). In group P, 162 genes located in the 500 kb upstream and downstream regions around genome-wide significant SNPs were considered candidate genes and were annotated to the Gene Ontology (GO) and Kyoto Encyclopedia of Genes and Genomes (KEGG) databases. These genes were enriched in 41 GO functions and 9 pathways. Several enriched pathways related to the host immune system were detected, such as the Wnt signaling pathway and the inflammatory mediator regulation of TRP channels pathway. *PRKCA*, *ADCY9*, *CAMK2B*, and *PRKCD* were involved in these pathways in group P (Table S4). Meanwhile, 131 significant SNPs were discovered in group N (*P < *3.1 × 10^−7^) (Table S3). In this group, 16 and 13 SNPs were enriched on GGA1 and GGA8, respectively (Fig. 3b and Table S3). Accordingly, the genes located 500 kb upstream and downstream of genome-wide significant SNPs in group N were screened. They were involved in GO functions, such as skeletal muscle tissue regeneration, ATP hydrolysis-coupled proton transport, and cellular response to retinoic acid (Table S4).

10.1128/mSystems.01192-20.4 TABLE S3 Significant SNPs (*P < *3.1 × 10^−7^) in groups P and N. Download Table S3, XLSX file, 0.03 MB. Copyright © 2021 Ding et al. 2021 Ding et al. This content is distributed under the terms of the Creative Commons Attribution 4.0 International license.

10.1128/mSystems.01192-20.5 TABLE S4 Candidate genes in groups P and N mapped to the GO and KEGG databases. Download Table S4, XLSX file, 0.02 MB. Copyright © 2021 Ding et al. 2021 Ding et al. This content is distributed under the terms of the Creative Commons Attribution 4.0 International license.

### Host genetic loci interacted with individual gut microbes.

**FIG 4:**
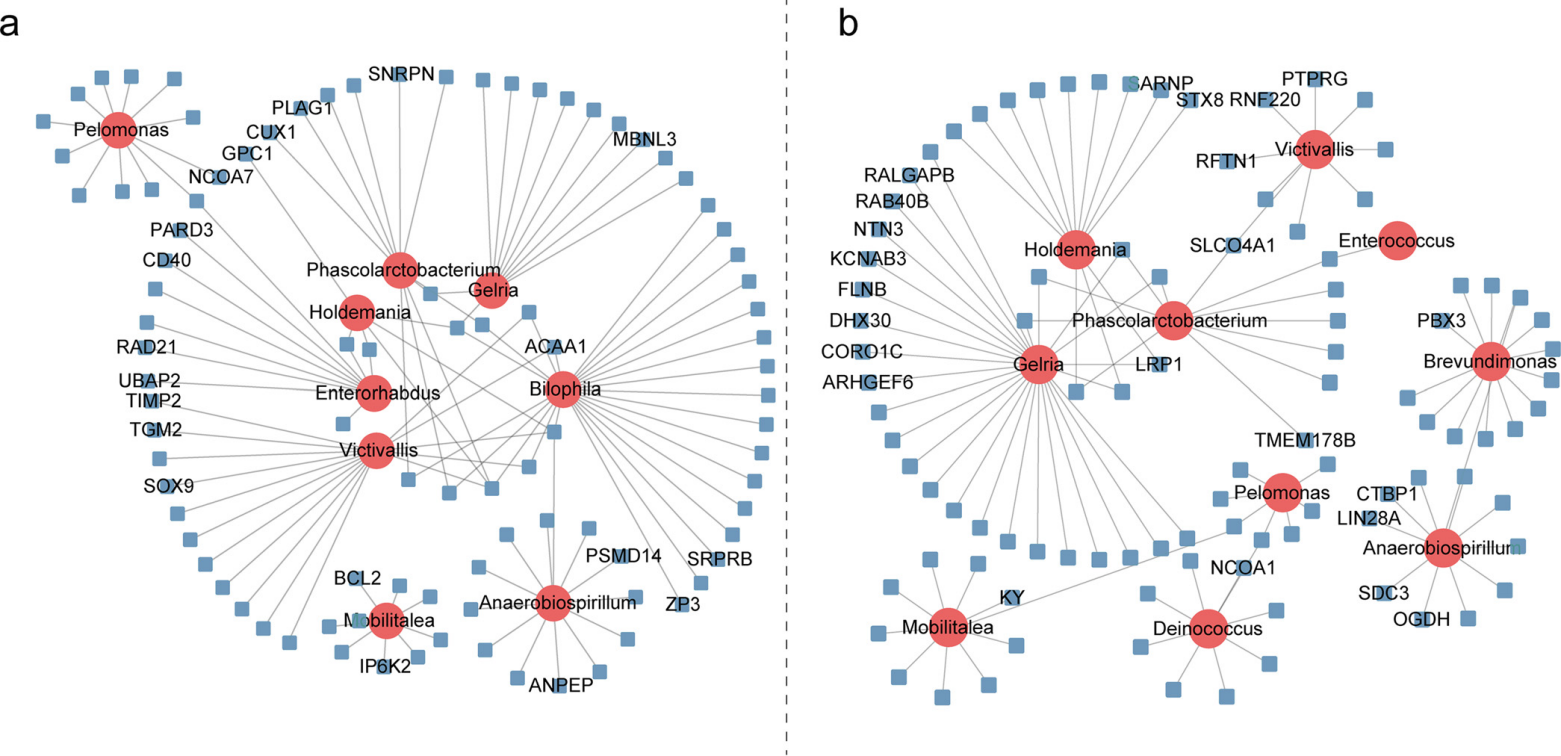
Association between host genetic variants and significantly different bacteria between groups P and N. (a) Significantly different microbes associated with the identified SNPs in group P. (b) Significantly different microbes associated with the identified SNPs in group N.

To identify host genes influencing the gut microbiota, mGWAS was performed using 159,272 SNPs identified from groups P and N. Detailed information on the experimental chickens, SNPs, operational taxonomic units (OTUs), and genera used for mGWAS is provided in Table S5. In group P, 1,219 SNPs were associated (*P < *6.28 × 10^−6^) with 177 bacterial genera (Table S6). The most significant association was noted between the SNP at 52,541,080 bp on GGA5 and *Facklamia* (*Firmicutes*). This SNP was close to *NUDT14* (Table S6). Ten of the 177 genera were more abundant in group P than in group N (*P < *0.05) (Table 1 and Fig. 4a). Of these, *Bilophila* (*Proteobacteria*) was associated with 25 genes, such as *SRPRB*, *ZP3*, and *ACAA1* (Table S6). *Victivallis* (*Lentisphaerae*) and *Phascolarctobacterium* were correlated with 16 and 11 genes, respectively (Fig. 4a). *Pelomonas*, which had a high abundance in group P, was found to be associated with 13 SNPs, one of which was located at the intronic region of *NCOA7*. Genes such as *TGIF1* and *TTLL12* were found to be close to the locus of this SNP. Moreover, *Anaerobiospirillum*, which was more enriched in group P, was associated with 13 SNPs. Of these, one SNP was located at the exonic region of *ANPEP* on GGA10. Another SNP was located at the intronic region of *PSMD14* (Fig. 4a). Furthermore, 12 of the 1,219 significant SNPs were distributed in the exonic region. Of them, 3 nonsynonymous SNPs located at *RARRES2*, *PIP5K1A*, and *TAP2* were associated with *Facklamia* and *Weissella*, which belonged to the order *Lactobacillales* (Table 2).

**TABLE 2:** The genus significantly associated with exonic single-nucleotide variants (SNVs) (*P < *6.28e−06) in groups P and N

Genus (phylum)	Chr	Position	Ref/alt^*b*^	Gene	*P* value
Group N					
*Gastranaerophilales_unidentified* (*Cyanobacteria*)	4	51596858	T/G	*CENPC*	2.37e−06
*Gastranaerophilales_unidentified* (*Cyanobacteria*)	4	51596775	A/G	*CENPC*	2.37e−06
*Gastranaerophilales_unidentified* (*Cyanobacteria*)	4	51596800	T/C	*CENPC*	2.37e−06
*Gastranaerophilales_unidentified* (*Cyanobacteria*)	4	51596764	T/C	*CENPC*	2.37e−06
*Coriobacteriaceae_Uncultured* (*Actinobacteria*)	5	24668248	G/A	*CHAC1*	3.06e−07
*Lachnospiraceae_FCS020* (*Firmicutes*)	7	22055862	G/C	*INHA*	3.26e−06
*Rothia* (*Actinobacteria*)	28	1675114	T/C	*LARP6L*	2.87e−06
*Ruminococcaceae_UCG.013* (*Firmicutes*)	33	4712604	C/T	*LRP1*	1.42e−06
*Lachnoclostridium_12* (*Firmicutes*)	4	88540606	A/G	*MAVS*	5.75e−06
*Kocuria* (*Actinobacteria*)	4	78807529	T/C	*MSX1*	2.69e−06
*Gallibacterium* (*Proteobacteria*)	20	3626488	G/A	*MYBL2*	3.48e−06
*Solobacterium* (*Firmicutes*)	9	15443945	T/C	*NMUR1*	2.53e−06
*Anaerosporobacter* (*Firmicutes*)	24	3538527	A/G	*SORL1*	1.07e−06
*Corynebacteriaceae_unidentified* (*Actinobacteria*)	24	434592	T/C	*SRPRA*	5.41e−06
*Kocuria* (*Actinobacteria*)	11	18715842	G/A	*SPG7*	4.78e−06
*Collinsella* (*Actinobacteria*)	2	477467	A/G	*SSPO*	1.78e−06
*Mollicutes_RF9_unidentified* (*Tenericutes*)	2	479493	T/C	*SSPO*	1.58e−06
*Victivallaceae_unidentified* (*Lentisphaerae*)	2	61480667	G/A	*MYLIP*	3.95e−06
*Ruminococcus_torques* (*Firmicutes*)	18	9864374	A/G	*SYNGR2*	5.38e−07
Group P					
*Uncultured_rumen_bacterium* (*Lentisphaerae*)	6	12687483	T/G	*CHST3*	4.20e−08
*Microbacterium* (*Actinobacteria*)	3	8282453	T/C	*STON1*	5.77e−07
*Prevotella_2* (*Bacteroidetes*)	3	104478227	T/C	*PREB*	8.11e−07
*Globicatella* (*Firmicutes*)	28	2108770	T/C	*AMH*	1.21e−06
*Slackia* (*Actinobacteria*)	6	16780551	A/G	*FUT11*	1.58e−06
*Lachnoclostridium_12* (*Firmicutes*)	4	45675385	T/C	*DMP1*	2.81e−06
*Anaerobiospirillum* (*Proteobacteria*)	10	20439699	T/C	*ANPEP*	2.81e−06
*Ruminiclostridium_5* (*Firmicutes*)	7	18219839	G/A	*GAD1*	2.99e−06
*Uncultured_bacterium* (*Firmicutes*)	2	64669929	A/G	*RREB1*	3.40e−06
*Microbacterium* (*Actinobacteria*)	2	458529	T/C	*RARRES2* ^*a*^	4.37e−06
*Facklamia* (*Firmicutes*)	25	269948	G/C	*PIP5K1A* ^*a*^	4.94e−06
*Weissella* (*Firmicutes*)	16	2602666	T/C	*TAP2* ^*a*^	4.75e−06

a Nonsynonymous SNVs at genes; the others are synonymous SNVs at genes.

b Ref: the allele in the reference genome, alt: any other allele found at that locus.

10.1128/mSystems.01192-20.6 TABLE S5 Numbers of experimental animals, SNPs, OTUs, and genera and the significant thresholds used for mGWAS. Download Table S5, DOCX file, 0.01 MB. Copyright © 2021 Ding et al. 2021 Ding et al. This content is distributed under the terms of the Creative Commons Attribution 4.0 International license.

10.1128/mSystems.01192-20.7 TABLE S6 Identification of host genetic variants associated with the gut microbiota. Download Table S6, XLSX file, 0.2 MB. Copyright © 2021 Ding et al. 2021 Ding et al. This content is distributed under the terms of the Creative Commons Attribution 4.0 International license.

In group N, 1,118 SNPs were prominently linked to 181 genera (Table S6). The most significant association was noted between the SNP located at 175,426,469 bp on GGA1 and *Brachybacterium* (*Actinobacteria*) (*P = *1.29 × 10^−11^). This SNP was located at the intergenic region between *RFC3* and *PDS5B* (Table S6). Moreover, the SNP located at the intronic region of *KY* was found to be associated with *Mobilitalea* (Fig. 4b). The abundance of *Mobilitalea* was higher in group N. *Brevundimonas* was associated with 14 SNPs, two of which were located at 10,120,154 bp and 10,120,159 bp on GGA17 at the intronic region of *PBX3* (Fig. 4b). The remaining SNPs associated with *Brevundimonas* were located at the intergenic regions of several immune-related genes, including *CCR7* and *PLEKHJ1* (Table S6). Furthermore, 19 synonymous SNPs were detected at the exonic region of 15 genes that were associated with 15 genera (Table 2). Among them, 4 SNPs in *CENPC* were associated with the abundance of *Gastranaerophilales_unidentified* (*Cyanobacteria*).

## DISCUSSION

In this study, we explored the relationship among host genetics, gut microbiome, and *S*. Pullorum infection in chickens. Microbiome comparison revealed that *S*. Pullorum infection in chickens altered the gut microbial composition, resulting in variation of the microbial metabolic function. The abundance of 39 bacterial genera differed between groups P and N. Moreover, compared to group ON, group OP showed a remarkable difference in microbial composition and a high abundance of potentially harmful bacteria. *Pelomonas* and *Brevundimonas* exhibited heritability in the offspring coming from *S*. Pullorum-infected chickens. *Pelomonas* has been reported to be the dominant bacterium in patients with serious inflammatory bowel disease (35). Similarly, an increased abundance of *Brevundimonas* has been found in the intestinal mucosa of patients with ulcerative colitis (36). These findings suggest that *S*. Pullorum infection disturbs the structure of the gut microbiota and the abundance of related microbes in infected individuals and their offspring.

In addition, the heritable gut microbiota was found to be influenced by host genetic variants. In group P, *Pelomonas* was associated with SNPs that were close to genes such as *TGIF1* and *TTLL12*. *TGIF1* promotes the endothelial cell inflammatory response in the gut of mice (37). *TTLL12* specifically inhibits the expression of the downstream genes of innate immunity pathways (38). These findings suggest that *TGIF1* and *TTLL12* interact with *Pelomonas* to affect intestinal homeostasis in *S*. Pullorum-infected chickens and cause symptoms such as diarrhea. In group N, the abundance of *Brevundimonas* was associated with 14 SNPs. These SNPs were close to several immune-related genes in the chicken genome. Among them, *CCR7* plays a critical role in controlling T-cell retention/egress to maintain intestinal homeostasis in mice (39). The interaction between *CCR7* and *Brevundimonas* may play a role in the maintenance of gut homeostasis in chickens.

In group P, 4 SNPs associated with gut microbial beta diversity were located at the intronic region of *MAPKKK3L*, which belongs to the *MAPKKK* family. *MAPKKK* can regulate several signal transduction pathways, including c-Jun NH2-terminal kinase, ERK, and nuclear factor-κB (NF-κB), by stimulating the Toll-like receptor (40–42). Moreover, it can stimulate immune cells, such as macrophages, dendritic cells, and neutrophils, to produce various chemokines, including gamma interferon (IFN-γ) and tumor necrosis factor alpha (TNF) (43). *MYD88*, located downstream of *MAPKKK3L* (less than 26 kb), is associated with susceptibility to *S*. Pullorum infection (44). Thus, *MAPKKK3L* may be a vital candidate gene associated with the gut microbiota in *S*. Pullorum-infected chickens. Therefore, the heritable bacteria *Pelomonas* and *Brevundimonas* and significant markers located on related genes, such as *TGIF1*, *TTLL12*, *CCR7*, and *MAPKKK3L*, could be used for the selection of resistant chickens and the elimination of pullorum disease.

## MATERIALS AND METHODS

### Animals and sampling.

The chickens (Xin Pudong chickens) used in the present study were obtained from the Animal Husbandry and Veterinary Research Institute, Shanghai Academy of Agricultural Science, Shanghai, China. None of the chickens had been treated with antibiotics. The wing venous blood and feces of 275 hens (52 weeks old; 140 in group N and 135 in group P) were collected. Ten positive roosters were mated with 10 positive hens, and 10 negative roosters were mated with 10 negative hens to obtain their respective offspring. All the chickens were maintained at the same location and fed the same diets. Eighty fecal samples of the offspring were collected at the age of 10 days (40 in group ON and 40 in group OP). In total, 355 fecal samples were collected and stored at −80°C. The protocols in the present study were approved by the Laboratory Animal Research (ILAR) guide for the care and use of laboratory animals at Shanghai Jiao Tong University, China.

### Rapid slide agglutination test.

*S*. Pullorum infections in chickens were diagnosed by *S*. Pullorum and *S*. Gallinarum polyvalent antigen rapid slide agglutination test reagents (product code 03.01.001.001; Beijing Zhonghai Biotech Co., Ltd., China). In brief, 50 μl of polyvalent antigen and 50 μl of venous blood were placed on a clean glass slide. The antigen and blood were thoroughly mixed and smeared into a circle of 2 cm on the glass slide. The samples were considered positive if 50% or more agglutination occurs in the mixture within 2 min. Samples without agglutination were deemed negative.

### 16S rRNA gene sequencing.

Microbiome DNA was isolated from the fecal samples using the Tiangen DNA stool minikit (number DP328; Tiangen, China) by following the manufacturer’s instructions. The extracted DNA was quantified on a NanoDrop spectrophotometer (Thermo Scientific). The DNA samples were stored at −20°C for further analysis. The V3-V4 regions of the 16S rRNA of all the fecal samples were amplified by PCR using barcoded fusion primers (forward primer 338F, ACTCCTACGGGAGGCAGCA; reverse primer 806R, GGACTACHVGGGTWTCTAAT). The PCR conditions were 98°C for 2 min; 98°C for 15 s; 55°C for 30 s and 72°C for 30 s, repeated for 30 cycles; and 72°C for 5 min. PCR amplicons were excised from a 1.5% agarose gel and purified using the QIAquick gel extraction kit (number 28706; Qiagen, Germany). Purified PCR products were combined at equal concentrations and used to construct a metagenomic library using the Illumina TruSeq sample preparation kit (Illumina) according to the manufacturer's protocol. Sequencing was performed by Shanghai Personal Biotechnology Limited Company (Shanghai, China) using the Illumina MiSeq sequencing platform (Illumina).

### Sequence quality control.

The Quantitative Insights Into Microbial Ecology (QIIME, v1.8.0) pipeline was employed to process the sequencing data, as described previously (45). In brief, raw sequencing reads with exact matches to the barcodes were assigned to the respective samples and identified as valid sequences. Low-quality sequences were filtered out according to the following criteria (46, 47): sequences with a length of <150 bp, average Phred scores of <20, contained ambiguous bases, and contained mononucleotide repeats of >8 bp. Chimeric sequences were removed using USEARCH (v5.2.236) in QIIME. Paired-end reads with an overlap longer than 10 bp between read 1 and read 2 and without any mismatch were assembled using FLASH (48).

### Microbial taxonomic annotation.

The filtered high-quality sequences were clustered into operational taxonomic units (OTUs) at 97% sequence identity using UCLUST (49). A representative sequence was selected from each OTU using default parameters. OTU taxonomic annotation was performed by BLAST searching the representative sequences set against the Silva database (50) using the best hit (51). An OTU table was further generated to record the abundance of each OTU in each sample and the taxonomy of OTUs. OTUs containing less than 0.001% of the total sequences across all the samples were discarded. To minimize the difference of the sequencing depth across samples, an averaged, rounded, rarefied OTU table was generated by averaging 100 evenly resampled OTU subsets under 90% of the minimum sequencing depth for further analysis.

### Microbiome comparison analysis.

Microbiome comparisons were performed between group N versus group P and group ON versus group OP. OTU-level alpha diversity indices, Chao1 richness estimator (52), and abundance-based coverage estimator (ACE) (53) were calculated using the OTU table in QIIME. Principal component analysis (PCA) was conducted based on the genus level compositional profiles and the plot drawn by R (54). Box plots and bar charts were created using SigmaPlot (55). Two-sided Welch’s *t* test and Benjamini-Hochberg false discovery rate (FDR) (*P < *0.05) (56) correction were used in two-group analysis. Microbial functions were predicted using PICRUSt (57). OTUs were mapped to the gg13.5 database at 97% similarity using the QIIME’s command “pick_closed_otus.” OTU abundance was automatically normalized using the 16S rRNA gene copy numbers from known bacterial genomes in Integrated Microbial Genomes (IMG). The predicted genes and their functions were aligned to the Kyoto Encyclopedia of Genes and Genomes (KEGG) database, and the differences among groups were compared using the STAMP software (58).

### Genotyping of populations from groups P and N.

The genomic DNA of 135 chickens in group P and 140 chickens in group N was extracted from their venous blood using the TIANamp blood DNA kit (number DP348; Tiangen, China). The DNA was used to construct double-digest genotyping-by-sequencing (dd-GBS) libraries (31), which were sequenced at Shanghai Personal Biotechnology Co., Ltd. The dd-GBS data were analyzed using the chicken genome (GRCg6a). The approach of single-nucleotide polymorphism (SNP) calling was consistent with that in our previous study (31). Next, quality control was performed for the genotyping data with call rate thresholds of ≥50%, minor allele frequency (MAF) of ≥5%, and Hardy-Weinberg equilibrium (HWE) *P* value of >1 × 10^−6^ using PLINK. A final set of 159,272 SNPs that passed the quality control assessment were used for further analyses.

### mGWAS for assessing the association between host genetic loci and gut microbial beta diversity.

The association between the gut microbial community and the host genetics was analyzed by performing mGWAS (59). In total, 159,272 SNPs were used as genotyping data, and a pairwise microbiome distance matrix of weighted UniFrac was used as the microbiome data. Mutations with adjusted *P* values that passed the genome-wide significance threshold (0.05/SNP number) were considered significant. SNPs were annotated using ANNOVAR (60), and the genes that contained significant SNPs were annotated by Gene Ontology (GO) and KEGG analysis using DAVID (61).

### mGWAS for assessing the effect of host genetic variants on gut microbial abundance.

To identify the genetic variants in groups P and N that were associated with the abundance of individual gut bacteria, a statistical test was performed for each association between SNPs and the taxa. The analysis was performed using the MiBioGen miQTL pipeline (62). In brief, the taxa that were detected in at least 10% of the samples were included; their relative abundance was log transformed and controlled for the effects of the first three genetic principal components. The taxa were treated as quantitative traits, and a linear regression model of their log-transformed relative abundance was adopted with Fisher’s test-based *P* value estimation. In total, 197 taxa were defined as a binary trait (absence/presence) using logistic regression with chi-square-based *P* value estimation. The binary and quantitative models were used for groups N and P, respectively. The genome-wide significance threshold for the association was set at 0.05/SNP number. The suggestive significance level was determined by 1/SNP number.

### Data availability.

Raw read sequences are publicly available in the Sequence Read Archive at National Center for Biotechnology Information (NCBI) under the BioProject accession number PRJNA679403.
