# Supplementary figures and images for: Heritable Gut Microbiome Associated with Salmonella enterica Serovar Pullorum Infection in Chickens
Source: mSystems. 2021 Jan 5;6(1):e01192-20. doi: 10.1128/mSystems.01192-20 (PMC7786134; doi:10.1128/mSystems.01192-20)

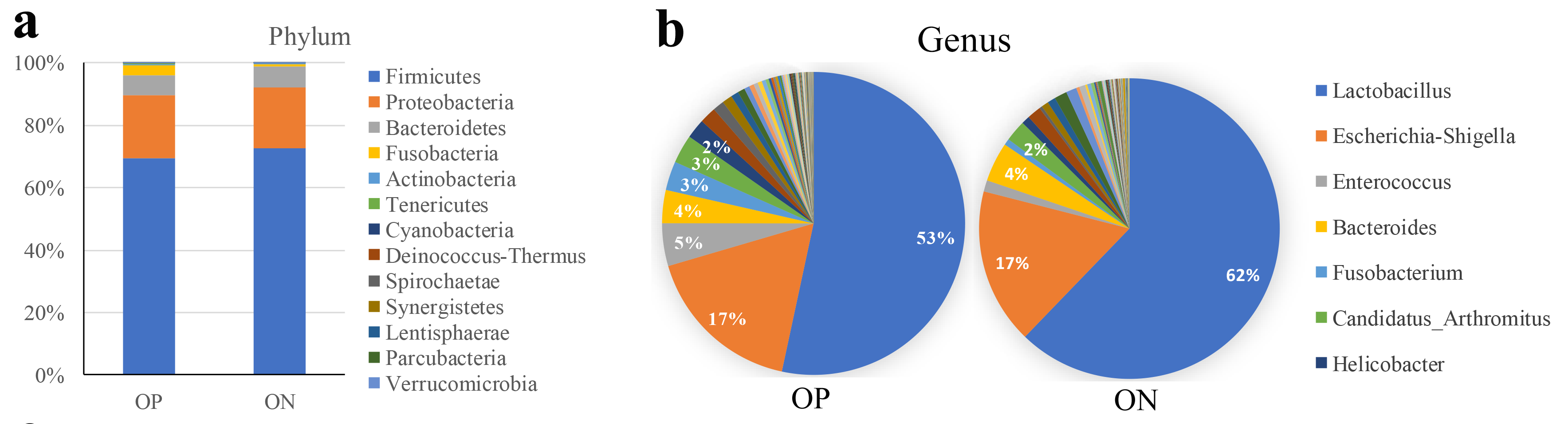

Supplement: FIG S1 [file mSystems.01192-20-sf001.tif]
