# Supplementary material for: Heritable Gut Microbiome Associated with Salmonella enterica Serovar Pullorum Infection in Chickens
Source: mSystems. 2021 Jan 5;6(1):e01192-20. doi: 10.1128/mSystems.01192-20 (PMC7786134; doi:10.1128/mSystems.01192-20)
Supplement: TABLE S1 [file mSystems.01192-20-st001.docx]

Table S1: Gut microbiota with significant difference between group OP and group ON at phylum level

| Phylum | ON-mean | OP-mean | p-value |
| --- | --- | --- | --- |
| *Parcubacteria* | 0 | 0.000022 | 0.000008 |
