# Supplementary material for: Heritable Gut Microbiome Associated with Salmonella enterica Serovar Pullorum Infection in Chickens
Source: mSystems. 2021 Jan 5;6(1):e01192-20. doi: 10.1128/mSystems.01192-20 (PMC7786134; doi:10.1128/mSystems.01192-20)
Supplement: TABLE S2 [file mSystems.01192-20-st002.docx]

Table S2: Gut microbiota with significant difference between group OP and group ON at the genus level.

| Genus | ON-mean | OP-mean | p-value |
| --- | --- | --- | --- |
| *Aegypius_monachus_(black_vulture)* | 0 | 0.000033 | 0** |
| *Alcaligenes* | 0.000033 | 0 | 0** |
| *Blattella_germanica_(German_cockroach)* | 0.000036 | 0.000001 | 0** |
| *Brevundimonas* | 0 | 0.000026 | 0** |
| *[Eubacterium]_rectale_group* | 0 | 0.000045 | 0** |
| *Lachnospiraceae_AC2044_group* | 0 | 0.000026 | 0** |
| *Novosphingobium* | 0 | 0.000028 | 0** |
| *Oscillospira* | 0.000003 | 0.000041 | 0** |
| *Prevotella_9* | 0 | 0.000042 | 0** |
| *Prevotellaceae_NK3B31_group* | 0 | 0.000027 | 0** |
| *Ruminococcaceae_UCG-011* | 0.000003 | 0.000046 | 0** |
| *Vagococcus* | 0.000049 | 0 | 0** |
| *Vibrionimonas* | 0.000001 | 0.00004 | 0** |
| *Acidovorax* | 0.000039 | 0.000004 | 0.000001** |
| *Aquabacterium* | 0.000007 | 0.000048 | 0.000001** |
| *Diaphorobacter* | 0.000026 | 0 | 0.000001** |
| *Asticcacaulis* | 0.000002 | 0.000031 | 0.000003** |
| *Megasphaera* | 0.000001 | 0.000025 | 0.000011** |
| *Stenotrophomonas* | 0.000021 | 0 | 0.000015** |
| *Lachnospiraceae_ND3007_group* | 0.000041 | 0.000008 | 0.000041** |
| *Cloacibacterium* | 0 | 0.000016 | 0.00012** |
| *Citrobacter* | 0.000027 | 0.000002 | 0.000121** |
| *Pelomonas* | 0.000007 | 0.000033 | 0.000318** |
| *Aeribacillus* | 0.000007 | 0.00003 | 0.000864** |
| *Lysinibacillus* | 0.000127 | 0 | 0.000999** |
| *Ochrobactrum* | 0.000054 | 0.000228 | 0.002** |
| *Legionella* | 0.000018 | 0.000002 | 0.00235** |
| *Kurthia* | 0.000021 | 0.000004 | 0.00258** |
| *Bacillus* | 0.000012 | 0.000051 | 0.005** |
| *Intestinibacter* | 0.000022 | 0.000005 | 0.0072** |
| *Corynebacterium* | 0.000002 | 0.000015 | 0.00731** |
| *Ureaplasma* | 0.000004 | 0.000382 | 0.00799** |
| *Lachnospiraceae_UCG-008* | 0.000021 | 0.000006 | 0.0118* |
| *Comamonas* | 0.000872 | 0 | 0.014* |
| *Moryella* | 0.000033 | 0.000014 | 0.0167* |
| *Jeotgalicoccus* | 0 | 0.000647 | 0.012* |
| *Enterococcus* | 0.0122 | 0.0458 | 0.022* |
| *Myroides* | 0.000369 | 0 | 0.032* |
| *gut_metagenome* | 0.000026 | 0.000049 | 0.0329* |
| *Peptococcus* | 0.000105 | 0.000021 | 0.035* |
| *Dysgonomonas* | 0.00002 | 0.000006 | 0.0414* |

(**p*<0.05, ***p*<0.01)
