# Supplementary material for: Heritable Gut Microbiome Associated with Salmonella enterica Serovar Pullorum Infection in Chickens
Source: mSystems. 2021 Jan 5;6(1):e01192-20. doi: 10.1128/mSystems.01192-20 (PMC7786134; doi:10.1128/mSystems.01192-20)
Supplement: TABLE S5 [file mSystems.01192-20-st005.docx]

Table S5: The numbers of experimental animals, SNPs, OTUs and genus, and the signiﬁcance threshold used for mGWAS.

| Group | Animal numbers | SNPs | Genome-wide significance | Suggestive significance | Genus | |
| --- | --- | --- | --- | --- | --- | --- |
|  |  |  |  |  | Quantitative | Binary |
| Positive | 135 | 159,272 | 3.10e-07 | 6.28e-06 | 214 | 196 |
| Negative | 140 | 159,272 | 3.10e-07 | 6.28e-06 | 214 | 197 |
